# RETRACTION: Effect of Prophylactic Negative Pressure Treatment for Post‐Surgery Groin Wounds Management in Vascular Surgery: A Meta‐Analysis

**DOI:** 10.1111/iwj.70468

**Published:** 2025-04-02

## Abstract

**RETRACTION:** XieR. , LiB. , and WenF. , “Effect of Prophylactic Negative Pressure Treatment for Post‐Surgery Groin Wounds Management in Vascular Surgery: A Meta‐Analysis,” International Wound Journal 20, no. 2 (2023): 269–277, 10.1111/iwj.13870.35818744 PMC9885472

The above article, published online on 11 July 2022, in Wiley Online Library (http://onlinelibrary.wiley.com/), has been retracted by agreement between the journal Editor in Chief, Professor Keith Harding; and John Wiley & Sons Ltd. Following an investigation by the publisher, all parties have concluded that this article was accepted solely on the basis of a compromised peer review process. The editors have therefore decided to retract the article. The authors did not respond to our notice regarding the retraction.

**RETRACTION:** R. Xie , B. Li , and F. Wen , “Effect of Prophylactic Negative Pressure Treatment for Post‐Surgery Groin Wounds Management in Vascular Surgery: A Meta‐Analysis,” International Wound Journal 20, no. 2 (2023): 269–277, 10.1111/iwj.13870.35818744 PMC9885472

The above article, published online on 11 July 2022, in Wiley Online Library (http://onlinelibrary.wiley.com/), has been retracted by agreement between the journal Editor in Chief, Professor Keith Harding; and John Wiley & Sons Ltd. Following an investigation by the publisher, all parties have concluded that this article was accepted solely on the basis of a compromised peer review process. The editors have therefore decided to retract the article. The authors did not respond to our notice regarding the retraction.

